# The Experience of Teleworking with Dogs and Cats in the United States during COVID-19

**DOI:** 10.3390/ani11020268

**Published:** 2021-01-21

**Authors:** Christy L. Hoffman

**Affiliations:** Department of Animal Behavior, Ecology, and Conservation, Canisius College, Buffalo, NY 14208, USA; hoffmanc@canisius.edu

**Keywords:** telework, COVID-19, human-animal interaction, pets, dogs, cats, well-being, work-life balance

## Abstract

**Simple Summary:**

During Spring 2020, the public health threat created by the novel coronavirus (SAR-CoV-2), commonly referenced as COVID-19, prompted employers across the United States to allow, encourage, or require their employees to work from home. This exploratory, survey-based study compared individuals’ perceptions of working from home and from their employer’s workplace, paying particular attention to how these experiences differed for individuals with and without dogs and/or cats. Participants reported they had more opportunities to socialize with other people and experienced less work-family conflict when they worked at the office. When working from home, participants indicated they spent more quality time with their pets and family members, and compared to those without dogs, dog owners were more likely to report socializing with others and getting a healthy amount of physical activity. Follow-up studies are needed to investigate whether these findings persist after the threat of COVID-19 abates and to see how characteristics of pet owners (e.g., personality, job type) and pets (e.g., species, age, behavior) impact any benefits or costs associated with being in the company of pets while teleworking.

**Abstract:**

In Spring of 2020, the novel coronavirus (SAR-CoV-2) prompted an unprecedented number of individuals across the United States to begin working from home. Prior research has identified both positive and negative impacts of teleworking on employee well-being, and this study built on that research to explore perceptions regarding how companion animals factor into the teleworking experience. Individuals who had experience working from home and from their employer’s office completed an online survey about those experiences. Participants reported spending more quality time with their companion animals and family members when they worked from home. Furthermore, when working from home, individuals with dogs were more likely than those without dogs to report they socialized with other people, got a healthy amount of physical activity, and took at least one 15-min walk during the workday. Some participants, particularly those in households containing both dogs and cats, indicated that their pets created distractions during the workday. Future studies can build on this research by investigating whether the findings persist once the novel coronavirus is no longer a threat, and by paying close attention to the characteristics of pets, owners, and household dynamics that may influence the effects of pet ownership on the teleworking experience.

## 1. Introduction

Technological advances have made it possible for millions of Americans to work remotely. Indeed, a 2019 report estimated that approximately 5 million individuals worked from home in the United States at least half the time [1]. During 2020, the number of teleworkers increased dramatically due the public health threat created by the novel coronavirus (SAR-CoV-2), commonly referenced as COVID-19. This virus prompted employers across the United States to allow, encourage, or require their employees to work from home beginning in March 2020. As recent statistics indicate that approximately 38% of households in the United States include a dog and 25% include a cat [2], for many individuals, the shift to teleworking meant spending more time within the proximity of companion animals.

Previous studies have found that working from home, often referenced as teleworking, has positive effects on employees. Some studies have even concluded that people are more productive when they work from home [3,4]. Additionally, teleworking is positively associated with objective ratings of performance [5]. Teleworking can also have favorable implications for an employee’s sense of work/family balance by freeing up commuting time for family activities and giving employees more autonomy and flexibility to help them meet both work and family demands [3,5].

Pets may enhance the experience of working from home. Their presence, as well as interactions with them, can increase humans’ endogenous oxytocin concentrations [6,7] and reduce heart rate and blood pressure [8,9]. In addition, they can buffer physiological responses to psychological challenges [10,11]. Allowing pets into traditional office environments has been shown to lessen work-related stress and create a more relaxed workplace climate [12,13]. Barker and colleagues found that self-reported stress levels tended to increase across the day when employees left their dogs at home, a pattern not observed when employees brought their dogs to the office [12]. The authors speculated that the stress buffering effects of pets may diminish as the time away from the pet increases, and that owners may become more concerned about their pets and miss them more as their time apart increases. Of course, when teleworking, pets and humans may spend little to no time apart. Thus, in the teleworking context, it is possible that pets may buffer stressors throughout the workday. 

Prior teleworking research indicates that the teleworking experience is not entirely positive. For instance, the lack of temporal and spatial separation between work and home can sometimes exacerbate work/family conflict [14], particularly since teleworking employees may extend their work into nontraditional work hours [15,16]. Such conflict is likely to occur unless supervisors proactively and explicitly encourage employees to set boundaries between work and family [17]. Without such boundaries, teleworking can create situations in which employees are likely to feel guilty, such as when they allow work to interfere with family time and when they take a break during the workday to tend to family issues [4]. Additionally, teleworking has the potential to be isolating because it reduces opportunities for employees to interact with colleagues [16]. 

Individuals’ relationships with their pets, as well as the types of activities in which they engage with their pets, may alleviate some of the problems that teleworking can exacerbate. For example, the routines, such as dog walking, that individuals establish with their pets may assist teleworkers in setting boundaries that denote a clear start and end to the workday. Because dogs commonly motivate their owners to get outside for walks [18], dogs also may enhance physical activity among teleworkers, particularly if the lack of a commute increases the amount of time teleworkers have available to engage in physical activity. Furthermore, dogs facilitate social interactions among people [19,20]. Thus, they may help offset feelings of social isolation that individuals may experience when working remotely, particularly if dog owners are regularly encountering some of the same acquaintances day after day while walking their dog.

By comparing data collected from pet owners and non-pet owners, all of whom had experience working both from home and from the office, this exploratory, survey-based study examined whether having a dog or cat at home was associated with individuals’ perceptions of the teleworking experience. As any effects of pets on the teleworking experience could only be fully appreciated by knowing how teleworking compared to working from the office, participants were also asked to report on the experience of working from the office. That is, in order to identify ways in which pets might affect the positive and negative aspects of working from home, it was important to determine how individuals’ perceptions of working from home and from the office differ. The questions about working from home and from the office focused on physical activity, socialization opportunities, distractions, and engagement with pets and family members. It was hypothesized that workplace location would affect these variables, and that pet ownership would be associated with the extent to which individuals reported socializing, exercising, and being distracted by others on days they worked from home.

As data were collected in June 2020, most participants reflected upon their experience of working from home as a result of COVID-19 and upon their experience of working from the office prior to the pandemic. It was predicted that dog owners would report engaging in physical activity, such as walking, more frequently than those without dogs, and that those with dogs and/or cats would engage in more activities with their pets on days they worked from home compared to days they worked from the office. Furthermore, it was predicted that, compared to non-pet owners, pet owners would score higher on a measure of job-related positive affect and lower on a measure of job-related negative affect in reference to working from home.

## 2. Materials and Methods

### 2.1. Participants

To ensure successful recruitment of dog owners, cat owners, and those with neither cats nor dogs, participants were sourced via a panel curated by Qualtrics. Sampling quotas were set to ensure data were collected from a minimum of 90 individuals who only had dogs, 90 who only had cats, 50 who had both dogs and cats, and 150 who had neither dogs nor cats. To participate in the panel, individuals had to be 18 years or older, employed full-time, and have had experience working both from home and from an employer’s office. In addition, they could not be self-employed at the time of survey completion. The survey was open to individuals both within and outside of the United States; however, responses from individuals who resided outside the United States (*n* = 14) were excluded from all analyses. Data were collected between 11 June 2020 and 22 June 2020. 

### 2.2. Measures and Procedures

When individuals clicked the link to access the survey, which was hosted by Qualtrics, they first viewed the informed consent document. Participants had to select a checkbox indicating their consent before continuing onto the next page of the survey. The first few questions that followed screened out individuals who did not meet the study’s inclusion criteria. Those who met the inclusion criteria answered questions about the frequency at which they currently worked for their employer from home and whether they were working from home, from their office, or from both locations prior to the social distancing measures put in place in Spring 2020 due to COVID-19. Participants also answered questions regarding whether the work they did from home was similar to the work they did at the office and whether they preferred working from home or the office.

Next, participants answered whether their household included a dog and/or a cat, and if any children and/or other adults lived in their household. Participants were then asked to indicate their level of agreement with a series of statements developed for the purposes of this study that were informed by existing literature on the effects of teleworking on well-being (e.g., “On the days I work from home, my work life and personal life interfere with each other”). Answer choices were presented on a 7-point Likert scale, with response options ranging from strongly disagree (1) to strongly agree (7). Those who had a pet were asked to rate their level of agreement with statements specific to how having a pet factored into their experience of working from home and from the office (e.g., “On the days I work at my employer’s workplace, I have the chance to spend quality time with household pets.”). Participants also answered questions regarding how often they stood up and took breaks during the workday, how much time they spent sitting, and how likely they were to take at least one 15-min walk. Statements related to working from home and working from the office were asked on different pages of the survey, and whether participants saw the statements about working from home before or after they saw the statements about working from the office was randomized, as was the order in which the statements on those pages were presented.

Participants rated how strongly they felt each of 10 emotions on days they worked from home and on days they worked from the office using a 5-point Likert scale, with answer choices ranging from strongly disagree (1) to strongly agree (5). These emotions were derived from the Job-Related Affective Well-Being Scale (JAWS) [21], and were used previously in a study that examined relationships between teleworking and affective well-being [22]. Five of the emotions were associated with positive affect (at ease, grateful, enthusiastic, happy, and proud), and five were associated with negative affect (bored, frustrated, angry, anxious, and fatigued). Whether participants were asked to rate the emotions in relation to working from home or working from the office first was randomized, as was the order in which the emotions were presented. Exploratory factor analysis on the 10 emotions supported the two-factor solution used by Anderson et al. (2014) [22] that separated the emotions into Positive Affective Well-Being (PAWB) and Negative Affective Well-Being (NAWB) subscales. Internal consistency reliability (α) was 0.83 for the PAWB subscale and 0.84 for the NAWB subscale.

Near the end of the survey, cat-owning participants indicated how likely they were to pet their cat for five or more consecutive minutes and play with their cat on days they worked from home and on days they worked at the office. Similarly, dog owners indicated how likely they were to pet their dog for five or more consecutive minutes, play with their dog, and walk their dog on days they worked from home and on days they worked at the office. The final section of the survey asked participants their zip code, their age, their race and ethnicity, and whether they lived in an urban, suburban, or rural community.

### 2.3. Data Analysis

All analyses were conducted using R version 3.6.3 [23]. Hierarchical binomial logistic regression modeling using the ‘lme4’ package version 1.1-26 [24] assessed factors associated with whether participants preferred working from home or the office. The null model contained only the dependent variable. The second model included participant gender and age, and the third included the addition of whether the household contained any of the following: Dogs, cats, other adults, children between 10 and 18 years old, children between 5 and 10 years old, and children under 5 years old. For assessing these models and all others described below, the ANOVA function tested whether there were significant differences between models. If there were no significant differences between models, the model with the lowest Akaike information criterion (AIC) value was chosen as the final model for each of the dependent variables. The ‘car’ package version 3.0-10 [25] was used to test for multicollinearity.

Answers to questions about workday activities and experiences on days at home and at the office were on an ordinal scale, and so hierarchical ordinal logistic regression modeling using the ‘ordinal’ package version 2019.12-10 [26] examined how demographic factors and workplace location impacted workday activities. Participant identification number was included as a random effect in these models because each participant answered questions about working from home and from the office. The null model only included the dependent variable and the random effect. The second model included participant gender and age, and the third model included the addition of workplace location.

Hierarchical ordinal logistic regression modeling also assessed how pets, other adults, and children in the household impacted activities and experiences had while working at home. The null model only included the dependent variable. The second model included participant age and gender, and the third included whether the household contained other adults, children, dogs, and/or cats.

For some ordinal logistic regression models, a test of the proportional odds assumption indicated that not all factors in the model met that assumption. In such cases, a median cutpoint was determined for the dependent variable, and scores on that measure were classified as being above or below/equal to the median. A binomial logistic regression then was performed.

A paired samples t-test compared PAWB and NAWB scores when working from home versus the office. Finally, hierarchical general linear models examined whether participant age, gender and the presence of pets and/or other humans in the home were associated with PAWB and NAWB scores on the days participants worked from home.

## 3. Results

The Qualtrics panel returned complete data from 454 individuals who resided in the United States. These individuals met the study’s eligibility criteria, completed the survey, and passed Qualtrics’ data screening process. Just over half of participants (*n* = 231) were female. Participants ranged in age from 19 years to 72 years (*M* = 41.3 years, *SD* = 11.5 years). One-third of the participants (*n* = 150) had one or more dogs and no cats; 21% (*n* = 97) had one or more cats and no dogs; 12% (*n* = 54) had at least one dog and one cat; and 34% (*n* = 153) had no dogs or cats. Table 1 provides additional details regarding sample characteristics, including race, ethnicity, community type, and the presence of other humans in the household.

Sixty-one percent of participants (*n* = 277) reported working exclusively from home at the time they completed the survey, but only 12% (*n* = 54) had worked exclusively from home in the months that immediately preceded the implementation of COVID-19-related policies (for more details, see Table 1). Sixty-seven percent of participants (*n* = 303) had worked from home within the prior month; 88% (*n* = 399) had within the prior three months; and 96% (*n* = 436) had within the prior six months (Table 1). Thirty-five percent of participants (*n* = 160) had worked from the office within the prior month; 61% (*n* = 276) had within the prior three months; and 89% (*n* = 405) had within the prior six months.

### 3.1. Work Location Preference in Relation to Pet Ownership and Household Members

Ninety-five percent of participants (*n* = 431) reported that the type of work they did at home was similar to or the same as the type of work they did at the office. Most participants indicated they preferred one workplace location to the other: 51% (*n* = 232) preferred working from home; 39% (*n* = 178) preferred working from the office; and 10% (*n* = 44) did not have a preference. When analyses were restricted to participants who did express a preference, the demographic model reflected that males were less likely than females to prefer working from home, and that there was no association between age and workplace location preference (Table 2). The model that included participant age, gender, and the presence of pets and people in the household did not have a significantly better fit than the demographic model (Δ*χ*^2^ = 3.97, Δ*df* = 6.0, *p* = 0.68), meaning that whether there were dogs or cats in the home did not impact workplace location preference.

### 3.2. Experiences of Working from Home versus the Office

Table 3 summarizes findings from the analyses that examined the effects of age, gender, and workplace location on participants’ experiences during the workday. Participants reported that they had more opportunities to socialize with other people on the days they worked from the office. In addition, they reported that work life and personal life were less likely to interfere with each other on days they worked from the office, and that family members were less likely to create distractions when working from the office. They also were less likely to indicate that they spent quality time with pets (β = −1.65, *SE* = 0.24, *p* < 0.001) and family members on days they worked from the office. In addition, they were less likely to play with or pet their dog on these days (play with dog: β = −1.15, *SE* = 0.29, *p* < 0.001; pet dog: β = −2.26, *SE* = 0.65, *p* < 0.001); however, workplace location had no statistically significant effect on how likely participants were to play with or pet their cat or walk their dog on workdays (play with cat: β = −0.77, *SE* = 0.39, *p* = 0.05; pet cat: β = −0.66, *SE* = 0.38, *p* = 0.08; walk dog: β = −0.50, *SE* = 0.32, *p* = 0.12).

Analyses that examined the associations between participant age, gender, and the presence of pets and other humans in the house and the experience of working from home are reported in Table 2. Participants who had dogs were more likely to report that work life and personal life interfered with each other (β = 0.91, *SE* = 0.23, *p* < 0.001), as were individuals with children under 10 years of age. Furthermore, those with dogs and/or young children were more likely to indicate that family members created distractions during the workday (dogs: β = 0.71 *SE* = 0.29, *p* = 0.01; children between 5 and 10: β = 0.65, *SE* = 0.29, *p* = 0.02).

When asked specifically about whether pets created distractions during the workday, 29% of individuals who had dogs but not cats (*n* = 43), 16% of individuals who had cats but not dogs (*n* = 15), and 46% of individuals who had both dogs and cats (*n* = 25) indicated their pets created distractions. The type of pet(s) owned was associated with the likelihood of indicating pets were a distraction during the workday (*χ*^2^(2, *n* = 301) = 16.69, *p* < 0.001). The standardized residuals revealed that fewer than expected individuals with cats and no dogs reported that their pets were distracting (*z* = −3.24), whereas a larger than expected number of individuals with both dogs and cats reported their pets were distracting (*z* = 3.40).

Although dogs created distractions for some participants when teleworking, their presence was also associated with behaviors that contribute positively to well-being. Participants with dogs reported socializing more with others on days they worked from home than did participants who did not have dogs (β = 0.62, *SE* = 0.22, *p* = 0.005). Participants who had dogs also reported getting more physical activity on days they worked from home when compared to those without dogs (β = 0.70, *SE* = 0.19, *p* < 0.001). In addition, dog owners were more likely than those without dogs to report taking at least one 15-min walk during the workday on days they worked from home (β = 0.93, *SE* = 0.20, *p* < 0.001).

### 3.3. Job-Related Affective Well-Being

PAWB and NAWB scores were calculated by averaging responses to the five items within each respective measure. PAWB scores ranged from 1 to 5, with 5 being indicative of extremely positive affect. The average PAWB score was 3.80 (*SD* = 0.77) when working from home and 3.75 (*SD* = 0.85) when working from the office. NAWB scores also ranged from 1 to 5, but in this case, 5 was indicative of extremely negative affect. The average NAWB score was 2.71 (*SD* = 1.00) when working from home and 2.79 (*SD* = 0.98) when working from the office. Paired samples t-tests indicated that neither PAWB scores nor NAWB scores differed significantly by workplace location (PAWB: *t* = 1.17, *df* = 453, *p* = 0.24; NAWB: *t* = −1.74, *df* = 453, *p* = 0.08). When analyses were restricted to when participants worked from home, neither PAWB scores nor NAWB scores were associated with the presence of dogs or cats in the home (Table 2).

## 4. Discussion

This study, which involved a within-subjects comparison of individuals’ perceptions regarding working from home and the office, provided numerous insights about the role dogs and cats play within the teleworking experience. Most participants expressed a workplace location preference, with just over half indicating they preferred working from home and 40% indicating they preferred working from the office. Neither the presence of dogs or cats nor the presence of other humans in the household predicted where participants preferred to work.

Just as no association existed between pet ownership and workplace location preference, no associations were found between pet ownership and participants’ positive and negative emotions regarding how their job made them feel when working from home. Even though survey results indicated that the experience of working from home with dogs and cats differs in some ways from the experience of teleworking without pets, pet ownership does not appear to be a driver of the emotions that are associated with teleworking. This finding was contrary to what had been hypothesized at the outset of this study.

While the data revealed no significant associations between pet ownership and workplace location preference, or between pet ownership and positive and negative affect when working from home, it is possible that dogs and cats may have enhanced the teleworking experience for some participants, had little to no effect for others, and worsened it for yet others. Characteristics of companion animals, such as pet type, age, and behavior, may influence the nature of the human–pet relationship. Prior studies have identified that human–pet attachment relationships vary across dyads and are associated with pets’ behavioral characteristics [27,28]. Furthermore, the human–animal bond is complex, with evidence suggesting that there are negative associations between particularly strong or weak human-animal bonds and human well-being [29,30,31].

Study participants reported having fewer opportunities to socialize with others on days they worked from home. The ability to engage with co-workers face-to-face can be beneficial to psychological well-being, particularly for individuals who do not have social partners at home. While technology enables remote workers to collaborate with each other, virtual interactions do not typically foster the establishment of the friendly, close relationships that tend to form in office environments [32]. Interestingly, survey responses indicated that dog ownership may enhance opportunities for individuals who work from home to socialize with others. That is, dog ownership may provide socialization opportunities that help offset the isolating nature of teleworking and the associated absence of opportunities to engage face-to-face with colleagues.

Specifically, dog owners reported they socialized more with others when teleworking compared to those without dogs. This may relate to the finding that dog owners were more likely than non-dog owners to report taking at least one fifteen-minute walk on days they worked from home and to report getting a healthy amount of physical activity on such days. These findings align with prior research that has shown that dog ownership is associated with more recreational walking [18,33]. Additionally, prior studies have demonstrated that dog walking enhances social interactions among community members [19,34]. As many businesses across the United States were closed to the public due to COVID-19 when data were collected, opportunities to engage with others outside of one’s household were largely restricted to outdoor activities, such as dog walking. Interestingly, dog owners indicated they were equally as likely to walk their dog on days they worked from home as on days they worked at the office, suggesting that dog walking was part of a routine that was unaffected by workplace location.

Dog walking may play a particularly important role in the physical health of individuals who work from home, especially since teleworking by its very nature reduces the number of steps individuals must take for utilitarian purposes [35]. That is, teleworking does not necessitate walking from home to the office and back, to and from a car in a parking lot, and/or to and from a bus or train stop. Furthermore, when working from home, the workday does not require one to walk to meetings in different parts of one’s office building or campus.

While opportunities to meet with co-workers are limited when teleworking, participants indicated they had more chances to spend quality time with companion animals, as well as with human family members, when working from home. Dog owners were more likely to spend time playing with and petting their dogs when they worked from home. The increased activity with dogs that working from home may not only strengthen the human–dog relationship, but may also confer psychological benefits to the human. There are positive associations between human–dog interactions and humans’ endogenous oxytocin concentrations [6,7]. In addition, petting and talking to dogs can reduce heart rate and blood pressure [8], as can spending time in a room with one’s pet cat [9]. Additionally, a dog’s or cat’s presence can attenuate cortisol levels and heart rate during psychological challenges [10,11].

The experience of spending more time with companion animals and other household members when teleworking was not entirely positive, as participants commonly indicated that pets created distractions. This was particularly true for individuals who had both dogs and cats in their home. Participants also reported that working from home exacerbated conflict between work life and personal life. While working from home frees up commuting time and often allows for a more flexible work schedule, teleworking conditions can muddle the boundaries between work time and family time. High levels of conflict between work and family are associated with high levels of exhaustion among teleworkers [15]. Teleworkers must carefully manage work-related and personal boundaries to minimize such conflict [36,37]. Some individuals with companion animals may find it challenging to maintain these boundaries because their animals are likely to be nearby at all times and may exhibit bothersome attention-seeking behaviors, such as whining, barking, and meowing excessively.

### Limitations and Future Directions

This survey-based study included a large, diverse sample of participants who varied in terms of their pet ownership status and who had experience working from both home and the office. The findings should be interpreted cautiously, however. The large number of statistical models employed in this study inflate the risk of Type I errors. Nevertheless, as the tables indicate, most pet-related findings that were significant at α < 0.05 would still have been significant had a more conservative α, such as α < 0.001, been selected.

Importantly, the data should also be considered with respect to the historical context in which data collection occurred. Nearly all participants reported having worked from both home and the office within the six months that preceded data collection; however, most had gained teleworking experience directly as a result of the workplace restrictions that businesses and governments instituted throughout much of the United States in Spring 2020 to minimize the spread of COVID-19. Thus, many participants may not have deliberately chosen to work from home under normal circumstances. Furthermore, many participants were likely to have been teleworking and caring for children simultaneously due to the pandemic and associated closures of schools and daycares. Therefore, participants in this study may have been more inclined to rate family members as distractors when teleworking than they would have under more typical circumstances. Follow-up studies are needed once the virus is no longer a public health threat and pandemic-related policies have been discontinued to determine if findings from this study generalize to teleworking with pets under more typical conditions.

While the within-subjects nature of the study design allowed for direct comparisons of individuals’ experiences working from home with their experiences of working from the office, the cross-sectional nature of the study meant that some participants had to recollect what they did and how they felt several months prior. For many participants, three months or more had passed since they had last worked at the office, thus increasing the likelihood of recall bias. That is, participants may not have evaluated an experience that occurred several months prior in the same way they would have evaluated that same experience promptly after it occurred. Studies that take a longitudinal approach over the span of days, weeks, or months and utilize ecological momentary assessments, as Barker et al. (2012) did [12], would provide more precise information about how pet owners and non-pet owners feel, what they do, and how productive they are when working from home and from the office. Adding physiological measures, such as cortisol or heart rate variability, would also provide valuable information regarding how dogs and cats may affect well-being when teleworking.

Although pet ownership was not associated with positive or negative affect when working from home, it is still possible that pets played an important role in the well-being of some teleworkers. The effects of dogs and cats on humans are not consistent across individuals. Prior research has demonstrated that the strength of the human–dog relationship, and the types of activities dogs and owners engage in together, moderate the effects that dogs have on human well-being and humans’ physiological responses to them [38,39,40]. Findings from the current study suggest that people not only have more opportunities to interact with their companion animals when they work from home, but also commonly take advantage of these opportunities. These interactions may strengthen the human–animal bond and the effects of pets on human well-being. Given that dog ownership was associated with greater physical activity when teleworking, it would be pertinent to examine whether dog owners are more inclined to participate in and complete employee-sponsored fitness challenges compared to non-dog owners. Furthermore, future research is needed to examine how increased engagement with pets impacts companion animal welfare and to establish best practices for ensuring healthy, mutually beneficial interactions between pets and individuals who telework.

Follow-up studies may find that the effects of pets on teleworkers’ well-being are impacted by human personality traits as well as by characteristics of the pet. For instance, a dog or cat that displays a high level of attention-seeking behaviors (e.g., whining, barking, or meowing excessively) may negatively affect the teleworking experience, especially for individuals who need a quiet home environment for frequent meetings via telephone or video conference, whereas more relaxed dogs or cats may have a more favorable impact. Such variation in human and pet characteristics may explain why some participants indicated their pets were distracting when working from home and others did not. Finally, future studies that focus on loneliness in relation to the teleworking experience may find that behavioral and physical characteristics of a dog enhance or hinder opportunities for social engagement with other humans while on dog walks. After all, the degree to which dog walking facilitates social interactions among community members is impacted by dog age, breed, and behavior [20,41].

## 5. Conclusions

For many, the presence of pets is one factor that distinguishes the experience of working from home from that of working from the office. This study specifically explored whether pet ownership was associated with differences in attitudes, psychological well-being, and physical activity for individuals when working from home versus from the office. Pet owners reported engaging more with their companion animals on the days they worked from home, and some individuals, particularly those who had both dogs and cats, indicated that their pets created distractions during the workday. In addition, compared to individuals without dogs, those with dogs reported socializing with other humans more and getting more physical activity on days they worked from home. Somewhat surprisingly, no relationships were found between pet ownership and positive or negative affect in relation to workplace location. Future studies that take a longitudinal approach, use ecological momentary assessments, and pay close attention to characteristics of pets and owners are needed to advance our understanding of ways that pets affect the teleworking experience and to identify best practices that ensure interactions between pets and individuals who telework are mutually beneficial.

## Figures and Tables

**Table 1:** Characteristics of the sample.

Variable	*N*
*Companion Animals*	
1 or more dogs, no cats	150 (33.3%)
1 or more cats, no dogs	97 (21.4%)
1 or more dogs and 1 or more cats	54 (11.9%)
No dogs or cats	153 (33.7%)
*Other Humans in Household*	
At least one other adult	217 (47.8%)
At least one child under 5 years	88 (19.4%)
At least one child 5–10 years	123 (27.1%)
At least one child 10–18 years	111 (24.4%)
No other humans	73 (16.1%)
*Race*	
American Indian/Alaska Native	8 (1.8%)
Asian	32 (7.0%)
Native Hawaiian or Other Pacific Islander	2 (0.4%)
Black/African American	69 (15.2%)
White	324 (71.4%)
Other	13 (2.9%)
Prefer not to say	6 (1.3%)
*Ethnicity*	
Hispanic/Latino	42 (9.3%)
Not Hispanic/Latino	407 (89.6%)
Prefer not to say	5 (1.1%)
*Community Type*	
Rural	61 (13.4%)
Suburban	175 (38.5%)
Urban	218 (48.0%)
*How much currently working from home?*	
Works exclusively from home every day of the work week	277 (61.0%)
Works from home multiple days per week, but goes to the office at least once per week	92 (20.3%)
Works from home occasionally, but less than one day per week	50 (11.0%)
Works from home one day per week, but goes to the office the other days of the week	34 (7.5%)
*Working from home prior to COVID-19?*	
Worked exclusively from the office	305 (67.2%)
Worked from the office part of the time	95 (20.9%)
Worked exclusively from home	54 (11.9%)
*How recently worked from home?*	
Within the past month	303 (66.7%)
Within the past 3 months	399 (87.9%)
Within the past 6 months	436 (96.0%)
More than 6 months ago	18 (4.0%)
*How recently worked from the office?*	
Within the past month	160 (35.2%)
Within the past 3 months	276 (60.8%)
Within the past 6 months	405 (89.2%)
More than 6 months ago	49 (10.8%)

**Table 2:** Regressions on outcome variables regarding the experience of working from home. Betas and standard errors are only included for covariates that were part of the final model. Bolded Akaike information criterion (AIC) values indicate which model was best.

Outcome Variable	*N*	AIC Null Model	AIC Demographic Model	AIC Housemates Model	Age	Gender (Male)	Other Adults	Children under 5	Children 5–10	Children 10–18	Dogs	Cats
					**Beta (SE)**	**Beta (SE)**	**Beta (SE)**	**Beta (SE)**	**Beta (SE)**	**Beta (SE)**	**Beta (SE)**	**Beta (SE)**
**Binary**												
Workplace location preference (Home)	454	563.3	**560.2**	568.2	0.02 (0.10)	−0.53 (0.20) **	x	x	x	x	x	x
Socialize with others	454	628.8	620.0	**609.6**	−0.04 (0.10)	0.31 (0.21)	0.07 (0.21)	0.13 (0.26)	0.56 (0.24) *	0.08 (0.24)	0.62 (0.22) **	0.44 (0.22) *
Work life and personal life interference	454	630.8	616.0	**577.5**	−0.11 (0.11)	0.19 (0.22)	0.12 (0.21)	0.72 (0.27) **	0.92 (0.25) ***	−0.20 (0.25)	0.91 (0.23) ***	0.42 (0.23)
Family creates distractions during workday	381	432.1	400.2	**381.1**	−0.21 (0.17)	0.97 (0.30) ***	−0.62 (0.28) *	0.47 (0.31)	0.65 (0.29) *	0.10 (0.30)	0.71 (0.29) *	0.20 (0.29)
**Ordinal**												
Time spent sitting	454	1389.17	**1357.02**	1358.10	0.44 (0.09) ***	−0.54 (0.17) **	x	x	x	x	x	x
Frequency stand up and move	454	1316.69	**1303.04**	1303.38	0.27 (0.08) **	−0.46 (0.17) **	x	x	x	x	x	x
Likelihood of taking 15-minute walk during break	454	1232.81	1193.25	**1175.38**	−0.29 (0.09) **	0.59 (0.19) **	−0.22 (0.18)	0.19 (0.23)	−0.006 (0.22)	0.02 (0.22)	0.93 (0.20) ***	0.10 (0.19)
Physical activity	454	1720.25	1696.91	**1686.79**	−0.10 (0.09)	0.59 (0.18) ***	−0.05 (0.17)	−0.27 (0.22)	0.17 (0.21)	0.06 (0.20)	0.70 (0.19) ***	0.30 (0.19)
Quality time with family	381	1318.58	1314.35	**1313.11**	0.13 (0.10)	0.29 (0.20)	−0.05 (0.22)	0.15 (0.24)	0.31 (0.22)	0.29 (0.22)	0.45 (0.20) *	0.16 (0.21)
Quality time with pets	301	**960.15**	961.13	960.90	x	x	x	x	x	x	---	---
Play with cat	151	462.87	**459.74**	463.63	−0.04 (0.17)	0.79 (0.30) **	x	x	x	x	---	---
Play with dog	204	**669.42**	672.38	676.60	x	x	x	x	x	x	---	---
Pet cat	151	519.41	**517.67**	522.18	0.00 (0.18)	0.71 (0.30) *	x	x	x	x	---	---
Pet dog	204	**681.01**	683.83	688.85	x	x	x	x	x	x	---	---
Walk dog	204	648.30	**643.57**	646.61	−0.23 (0.15)	0.73 (0.27) **	x	x	x	x	---	---
*Gaussian*												
PAWB	454	1054.9	**1050.9**	1055.0	−0.03 (0.04)	0.19(0.07) **	x	x	x	x	x	x
NAWB	454	1295.4	1285.9	**1285.8**	−0.14 (0.05) **	0.06 (0.10)	−0.11 (0.10)	−0.07 (0.12)	0.14 (0.11)	−0.25 (0.11) *	0.14 (0.10)	0.16 (0.10)

* *p* < 0.05, ** *p* < 0.01, *** *p* < 0.001, SE = standard error, AIC = Akaike information criterion, PAWB = Positive Affective Well-Being, NAWB = Negative Affective Well-Being, x = variable not included in the final model, --- = variable excluded from the models tested.

**Table 3:** Regressions on outcome variables in relation to age, gender, and workplace location. Betas and standard errors are only included for covariates that were part of the final model. Bolded AIC values indicate which model was best. Note that *N* represents the number of data points analyzed; participant identification number was treated as a random effect in this model to account for each participant providing information about working from home and from the office.

Outcome Variable	*N*	AIC Null Model	AIC Demographic Model	AIC Location Model	Age	Gender (Male)	Location (Office)
					**Beta (SE)**	**Beta (SE)**	**Beta (SE)**
**Binary**							
Socialize with others	907	1243.9	1245.0	**1141.9**	0.03 (0.10)	0.32 (0.19)	1.68 (0.20) ***
Family creates distractions during workday	762	1008.0	972.3	**903.8**	−0.56 (0.16) ***	1.53 (0.32) ***	−1.75 (0.25) ***
Quality time with family	762	1004.9	991.6	**944.0**	0.06 (0.12)	0.94 (0.24) ***	−1.30 (0.21) ***
Quality time with pets	602	822.3	815.6	**753.3**	0.23 (0.13)	0.67 (0.25) **	−1.65 (0.24) ***
Play with cat	302	346.7	331.4	**329.3**	−0.36 (0.42)	3.00 (0.88) ***	−0.77 (0.39)
Play with dog	408	540.1	539.4	**523.8**	−0.22 (0.23)	0.88 (0.44) *	−1.15 (0.29) ***
Pet cat	302	318.1	310.9	**309.7**	−0.24 (0.35)	1.91 (0.67) **	−0.66 (0.38)
Pet dog	408	448.7	450.6	**433.4**	−0.13 (0.48)	0.54 (0.92)	−2.26 (0.65) ***
Walk dog	408	468.6	462.3	**461.8**	−0.81 (0.37) *	1.58 (0.70) *	−0.50 (0.32)
**Ordinal**							
Time spent sitting	908	2675.68	**2658.99**	2659.95	0.38 (0.11) ***	−0.56 (0.21) **	x
Frequency stand up and move	908	2526.07	2495.85	**2495.81**	0.51 (0.11) ***	−0.79 (0.22) ***	0.19 (0.13)
Likelihood of taking 15-minute walk during break	908	2305.00	2249.02	**2248.43**	−0.66 (0.13) ***	1.52 (0.26) ***	−0.22 (0.14)
Physical activity	908	3390.66	**3363.16**	3365.16	−0.14 (0.08)	0.88 (0.17) ***	x
Work life and personal life interference	908	3422.46	3386.05	**3371.59**	−0.45 (0.10) ***	0.87 (0.20) ***	−0.50 (0.13) ***

* *p* < 0.05, ** *p* < 0.01, *** *p* < 0.001.

## Data Availability

The data presented in this study are available on request from the author.
